# Downregulation of miR-3568 Protects Against Ischemia/Reperfusion-Induced Cardiac Dysfunction in Rats and Apoptosis in H9C2 Cardiomyocytes Through Targeting TRIM62

**DOI:** 10.3389/fphar.2020.00017

**Published:** 2020-02-13

**Authors:** Xin Li, Xin Wang, Yuan-sheng Liu, Xiao-dong Wang, Jian Zhou, Hua Zhou

**Affiliations:** ^1^Department of Cardiovascular Medicine, Shanghai East Hospital, Tongji University School of Medicine, Shanghai, China; ^2^Department of Cardiovascular Medicine, Ji'AN Hospital, Shanghai East Hospital, Ji'ani, China

**Keywords:** ubiquitination, ischemia reperfusion, miR-3568, TRIM62, apoptosis, STAT3

## Abstract

microRNA-3568 (miR-3568) has been reported to be associated with atherosclerosis. Only few data describe the expression and underlying mechanism of miR-3568 in regulating cardiac ischemia–reperfusion (I/R) injury such as apoptosis. In this study, we therefore sought to investigate the potential function of miR-3568 in simulated I/R-induced apoptosis in H9C2 cardiomyocytes and related signaling pathways involved. Flow cytometry was performed to examine the cell apoptosis. The expression of miR-3568, Survivin, Bcl-2, ERK, JNK, p38, AKT, and STAT3 was measured by western blot and quantitative real-time PCR. The correlation between TRIM62 and p-STAT3 was measured by co-immunoprecipitation and ubiquitination. We found that miR-3568 expression in simulated I/R-induced H9C2 cardiomyocytes was increased in a time-dependent manner. miR-3568 mimic transfection in H9C2 cardiomyocytes significantly enhanced cell apoptosis, decreased the expression of Bcl-2 and Survivin, and activated STAT3 signaling, which were reversed by miR-3568 inhibitor. The direct interaction between miR-3568 and the 3′-untranslated region (UTR) of TRIM62 mRNA was confirmed by dual-luciferase reporter assay. TRIM62 overexpression or AG490, a selective inhibitor of JAK2/STAT3 significantly, significantly inhibited I/R and miR-3568 mimic induced cell apoptosis and STAT3 activation. TRIM62 was found to interact with and induce ubiquitination of p-STAT3. The facilitating role of miR-3568 in I/R injury was also observed in our *in vivo* rat models. In conclusion, our study suggests that miR-3568 promotes simulated I/R-induced apoptosis in H9C2 cardiomyocytes through targeting TRIM62.

## Introduction

Prolonged ischemia can lead to irreversible damage of myocardial cells, and timely reperfusion is the only effective measure to rescue ischemic myocardium. However, when the ischemic tissues receive the blood after reperfusion, they will produce more serious damage than ischemia including cardiomyocyte apoptosis and necrosis, cardiac dysfunction, and malignant arrhythmia, which is called ischemia–reperfusion (I/R) injury (Li et al., 2015a; Amoni et al., 2017; Lin et al., 2018). The main mechanisms of I/R injury include calcium overload, energy metabolism disturbance, autophagy, inflammatory cell infiltration, oxidative stress, cardiomyocyte apoptosis, and endothelial dysfunction (Chao et al., 2006; White et al., 2016; Zhang et al., 2016), involving various cellular mechanisms such as AMPK, MAPKs, AKT, and NF-κB signaling pathways (Bi et al., 2018; Feng et al., 2018; Lin et al., 2018; Yao et al., 2018). Myocardial I/R injury mainly results in four types: myocardial stunning, reperfusion arrhythmia, no reflow of blood vessels, and myocardial cell death that is the most serious form of reperfusion injury mainly including myocardial necrosis and apoptosis (Yellon and Hausenloy, 2007). The level of apoptosis reflects the severity of I/R injury to a certain extent. Inhibiting the occurrence and progress of apoptosis may prevent or alleviate I/R injury (Feng et al., 2018; Kosuru et al., 2018). Therefore, it is important to find effective measures to prevent against myocardial apoptosis after reperfusion.

MicroRNA (miRNA, miR) is an endogenous, small non-coding single strand RNA molecule, which is composed of 21–23 nucleotides and has a function of regulating gene expression after transcription by degradation or translation inhibition, and therefore plays an important role in the regulation of cell cycle, metabolism, growth, and disease progression. miRNA play a crucial role in cardiac development and homeostasis and that miRNA expression is altered in the diseased heart (Bartel, 2004; Chistiakov et al., 2016). To examine the role of miRNAs during I/R in the heart, we profiled the expression of several miRNAs after I/R injury. One interesting candidate was miR-3568. Previous studies found that miR-3568 was up-regulated in serum and liver in the rat with alcoholic steatohepatitis and associated with apoptotic process, MAPK signaling pathway (Chen et al., 2013), and its expression was also increased in matrix vesicles (MV) compared with vascular smooth muscle cell (VSMC) in the rats with chronic kidney disease, suggesting that the role of miR-3568 in vascular calcification and/or MV formation (Chaturvedi et al., 2015). The target genes of miR-3568 was involved in the MAPK signaling pathway and regulated osteoblast apoptosis (Chen et al., 2016). Although miR-3568 was up-regulated in the spinal cord of rats after I/R injury (Li et al., 2015b; Chen et al., 2019a), the potential function of miR-3568 in myocardial I/R injury has not been fully understood.

Signal Transducers and Activators of Transcription (STATs) is an important transcription factor containing seven members, STAT1–STAT4, STAT5a, STAT5b, and STAT6. STATs along with its upstream signaling Janus kinase family (JAK1, JAK2, JAK3, TYK2) are widely involved in many biological effects, such as cell stress, growth, proliferation, differentiation and apoptosis, and plays an important role in myocardial I/R injury and ischemic preconditioning myocardial protection mechanism (Barry et al., 2007). Studies show that JAK2/STAT3 activation contributes to cell apoptosis following cerebral and cardiac I/R (Zhang et al., 2011; Hu et al., 2017). However, the regulation of STAT3 signaling pathway remains unclear. Given the important role of STAT3 signaling in the process of cardiac I/R injury, we investigated potential function of miR-3568 in simulated I/R-induced H9C2 cardiomyocyte injury and that whether miR-3568 regulated cardiac I/R injury *via* the STAT3 signaling was also explored. Surprisingly, we observed that miR-3568 up-regulation was found in simulated I/R-induced H9C2 cardiomyocytes, and miR-3568 promoted simulated I/R-induced cell apoptosis *via* the STAT3 signaling pathway. TRIM62 (tripartite motif containing 62) as a target of miR-3568 inhibited miR-3568 overexpression induced cell apoptosis and STAT3 activation through ubiquitination of p-STAT3.

## Materials and Methods

### Simulated I/R Protocol *In Vitro*

H9C2 cardiomyocytes were cultured in DMEM containing 10 mM glucose, 1% penicillin–streptomycin (Solarbio, Beijing, China) and 10% fetal bovine serum (Gibco BRL, Grand Island, NY, USA) and incubated in a 37°C incubator with 5% carbon dioxide (CO_2_) for 24 h. Then H9C2 cardiomyocytes were transferred into sugar- and serum-free DMEM, incubated in 5% CO_2_ and 1% O_2_ at 37°C for 3 h, and then transferred into DMEM containing 10 mM glucose and 10% fetal bovine serum and incubated for 3, 6, 12, or 24 h for simulated I/R model.

### Cell Transfection

H9C2 cardiomyocytes were seeded in the six-well plate for 24 h and that with 50 nM miR-3568 mimic (sequence: 5′-UGUUCUUCCCGUGCAGAAGCAG-3′), miR-3568 inhibitor (sequence: 5′-CUGCUUCUGCACGGGAAGAACA-3′), or relative negative controls (NC) transfection for 6 h were performed using Lipofectamine 2000 (Invitrogen, Carlsbad, CA, USA) according to the instruction of the manufacturer. TRIM62 ectopic expression lentiviral vector was constructed by integrating the coding sequence (CDS) of TRIM62 into pLVX-Puro. The primers used to synthesize the CDS were as follows: forward: 5′-GCGAATTCATGGAGGAGAACAATGACT-3′; reverse: 5′-CGGGATCCCTGAATCCATATTGTGTTT-3′. 293T cells were seeded in the six-well plate and that with pLVX-Puro-TRIM62 or blank pLVX-Puro transfection for 4–6 h were performed using Lipofectamine reagent according to the manufacturer’s protocol. Viruses were collected after 48 h transfection and were used to infect H9C2 cardiomyocytes. The blank pLVX-Puro (Vector) was used as NC.

### Flow Cytometry Assay

H9C2 cardiomyocytes were seeded in six-well plate and maintained at 37°C for 24 h. Cells simulated ischemia for 3 h and reperfusion for 24 h were centrifuged at 1,000×g for 5 min and incubated with 5 μl Annexin V-FITC for 15 min and 5 μl PI for 5 min at 4°C. Cell apoptosis was analyzed on flow cytometer (Becton-Dickinson FACS Calibur, San Joes, CA, USA).

### Dual-Luciferase Reporter Assay

H9C2 cardiomyocytes were seeded in six-well plate, cultured in an incubator with 5% CO_2_ at 37°C for 24 h, and then co-transfected with 1.5 µg pGL3-basic plasmid containing 3′-untranslated region (3′-UTR) of WT (containing miR-3568 binding site) or MT (mutated miR-3568 binding site) TRIM62, miR-3568 mimic, or miR-3568 inhibitor at 37°C for 6 h using Lipofectamine 2000 (Invitrogen) following the manufacturer’s protocol. Forty-eight hours after transfection 100 µl of luciferase assay reagent and 10 µl of Stop&Glo reagent were added into the H9C2 cardiomyocytes. Luciferase activity (Firefly/Renilla) was measured with the Dual-Luciferase Reporter assay system (Promega, Madison, WI, USA) according to the manufacturer’s protocol.

### Quantitative Real-Time PCR

Total RNA was isolated and purified using Trizol reagent (Invitrogen) according to the manufacturer’s protocol. TRIM62, Bcl-2, and Survivin mRNA expressions were assessed using Applied Biosystems Prism 7300 sequence detection system with Maxima SYBR Green/ROX qPCR Master Mix according to the manual, using GAPDH as an internal normalized reference. The primers used were as follows: TRIM62 (XM_232757.9): forward 5′-TGGGTGTCTTCCTGGACTATG-3′; reverse 5′-TTAGATGCGGACCGTGTTG-3′. Survivin (NM_022274.1): forward 5′-TGGCTGCGCCTTCCTTACAGTC-3′; reverse 5′-AGTGGCTTAGCCGTGGCATGTC-3′. Bcl-2 (NM_016993.1): forward 5′-GATAACCGGGAGATCGTG-3′; reverse 5′-GGCTGGAAGGAGAAGATG-3′. GAPDH (NM_017008.4): forward 5′-GGAGTCTACTGGCGTCTTCAC-3′; reverse 5′-ATGAGCCCTTCCACGATGC-3′. Stem-loop real-time RT-PCR was carried out to analyze miRNA expression as previously described (Zhu et al., 2017). miR-3568 or 5S specific forward primer were used as follows: miR-3568, 5′-ACACTCCAGCTGGGTGTTCTTCCCGTGCAG-3′; 5S, forward 5′-AGGTGGTCTCCCATCCAAGT-3′; reverse 5′-CTACGGCCATACCACCCTGAAC-3′. 5S RNA was used as a miRNA internal control. The relative quantification was calculated using 2^−ΔΔCt^ cycle threshold method.

### Western Blotting

Total protein was extracted from H9C2 cardiomyocytes and myocardium with RIPA lysis buffer and then centrifuged at 12,000×g for 20 min at 4°C. Then, we separated the proteins using 10% SDS-PAGE and transferred them to a polyvinylidene fluoride membrane (Pall, Port Washington, NY, USA). Blots were blocked by using 5% non-fat milk for 1 h, and then incubated with TRIM62 (SAB1407634; 1:300, Sigma-Aldrich, St. Louis, MO, USA), Bcl-2 (Sc-492; 1:400, Santa Cruz Biotechnology, Inc., Santa Cruz, CA, USA), p-STAT3 (ab76315; 1:5000, Abcam, Cambridge, MA, USA), Survivin (#2808; 1:1000), p-ERK1/2 (#9101; 1:1000), ERK1/2 (#9102; 1:1000), p-JNK1/2 (#9255; 1:2000), JNK1/2 (#9252; 1:1000), p-p38 (#9211; 1:1000), p38 (#9212; 1:1000), p-AKT (#9271; 1:1000), AKT (#9272; 1:1000), STAT3 (#9139; 1:2000), or GAPDH (#5174; 1:2000) primary antibody at 4°C overnight. Survivin, p-ERK1/2, ERK1/2, p-JNK1/2, JNK1/2, p-p38, p38, p-AKT, AKT, STAT3, and GAPDH primary antibodies were obtained from Cell Signaling Technology (Danvers, MA, USA). Afterwards, the horseradish peroxidase-conjugated (HRP)-labeled goat anti-mouse (A0216), donkey anti-goat (A0181), or goat anti-rabbit IgG (A0208) secondary antibodies (1:1000, Beyotime Biotechnology, Shanghai, China) were further used for 2 h at 25°C. Blots were visualized with chemiluminescence and densitometric analysis was operated by a LAS 3000 imaging system (Fujifilm, Tokyo, Japan).

### Co-Immunoprecipitation and Ubiquitination Analysis

After H9C2 cardiomyocytes transduced with pLVX-Puro-TRIM62 or blank pLVX-Puro vector, the protein was extracted and treated with Protein A/G PLUS-Agarose (Santa Cruz Biotechnology, USA) for 1 h and incubated with anti-p-STAT3 (Abcam; ab76315; 1:5000), anti-TRIM62 (Sigma; SAB1407634; 1:300), or normal IgG (Santa Cruz Biotechnology; sc-2027; 1:1000) antibody at 4°C overnight, and the immunocomplexes were then associated with protein A-sepharose at 4°C for 2 h and centrifuged at 1,000×g at 4°C for 5 min. Anti-p-STAT3 (Abcam; ab76315; 1:5000) and anti-ubiquitin (Abcam; ab7780; 1:2000) antibodies were used for western blot analysis.

### Cardiac I/R Model in Rats

Cardiac I/R model in 10- to 12-week-old male rats (Sprague Dawley; Charles River, Morrisville, NC, USA) was performed as described previously by using a slipknot to ligate left anterior descending coronary artery (LAD) (Malka et al., 2016). Rats were anesthetized with a combination of 87 mg/kg ketamine and 13 mg/kg xylazine *via* intramuscular injection, intubated and mechanically ventilated at a rate of 80–90 cycles/min with a tidal volume of 1–2 ml/100 g. After 20 min of LAD ligation and reperfusion was allowed for 24 h or 2 weeks. The control rats underwent the same procedure except fastening the suture which was around the LAD. miR-3568 inhibitor (50 mg/kg/d) or miR-3568 NC was injected manually into the left ventricular anterior wall (four injections, 30 µl each, interspersed with 4′30″ between each injection) 24 h before I/R. The chest was closed after injection and the rat was allowed to recover. There were six rats in each group. For direct cardiac function evaluation, hemodynamic measurements were recorded and calculated with a pressure volume catheter (SPR-838, Millar Instruments, Houston, TX, USA) using the Millar pressure-volume system (MPVS-300, Millar Instruments) as previously described (Malka et al., 2016). Echocardiographic measurements were performed using a 7.5-MHz phased-array transducer connected to a sector scanner (SONOS 7500, Philips Medical Systems, Andover, MA) as previously described (Ma et al., 2017). Echocardiography cines were obtained according to the American Society of Echocardiography guidelines (Arias et al., 2013). When the experiment ended, rats were anaesthetized with ketamine (50 mg/kg) *via* intraperitoneal injection prior to be placed in a euthanasia chamber for 5 min that was filled with 100% diethyl ether. The myocardium were harvested, stained with hematoxylin and eosin (HE) or Masson′s Trichrome kit (Sigma), or incubated with terminal-deoxynucleoitidyl transferase mediated nick end labeling (TUNEL) as previously described (Luo et al., 2015; Qu et al., 2015; Xu et al., 2015). Rats were bred at our animal facility according to National Institutes of Health guidelines. The present study was performed in strict accordance with the guidelines on ethical care for experimental animals and approved by the Animal Research Committee of Shanghai East Hospital.

### Statistical Analysis

Each experiment was performed in triplicate unless stated otherwise, and the data were presented as the mean ± standard deviation (SD). Statistical analyses were carried out with the GraphPad Prism 5.0 software using one-way or two-way analysis of variance followed by Bonferroni post-test. P < 0.05 was considered to indicate a statistically significant difference.

## Results

### miR-3568 Expression Was Increased in Simulated I/R-Induced H9C2 Cardiomyocytes

**Figure 1:**
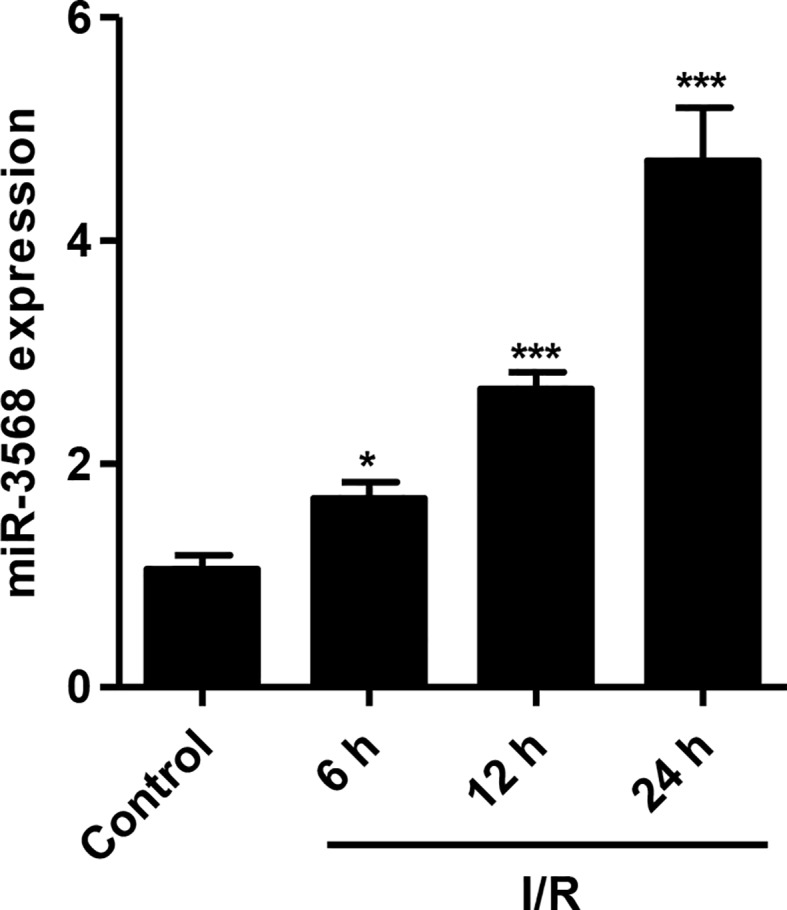
MicroRNA-3568 (miR-3568) expression in simulated ischemia–reperfusion (I/R)-induced H9C2 cardiomyocytes. H9C2 cardiomyocytes were simulated ischemia for 3 h and reperfusion for 6, 12, and 24 h, and the expression of miR-3568 was measured by quantitative real-time PCR (n = 3). Statistical analyses were carried out using one-way analysis of variance followed by Bonferroni post-test. *P < 0.05, ***P < 0.001 compared with control.

To explore the effect of miR-3568 on simulated I/R-induced cell injury in H9C2 cardiomyocytes, the miR-3568 expression in H9C2 cardiomyocytes simulated ischemia for 3 h and reperfusion for 6, 12, and 24 h was measured. As shown in **Figure 1**, miR-3568 expression in simulated I/R-induced H9C2 cardiomyocytes was increased by 67%, 169.8%, and 376.4% at 6, 12, and 24 h compared with that in control H9C2 cardiomyocytes, respectively. Therefore, we suggest that miR-3568 may associate with the simulated I/R injury in H9C2 cardiomyocytes.

### miR-3568 Mimic Enhanced Simulated I/R-Induced Cell Apoptosis and Decreases in The Survivin and Bcl-2 Expression

**Figure 2:**
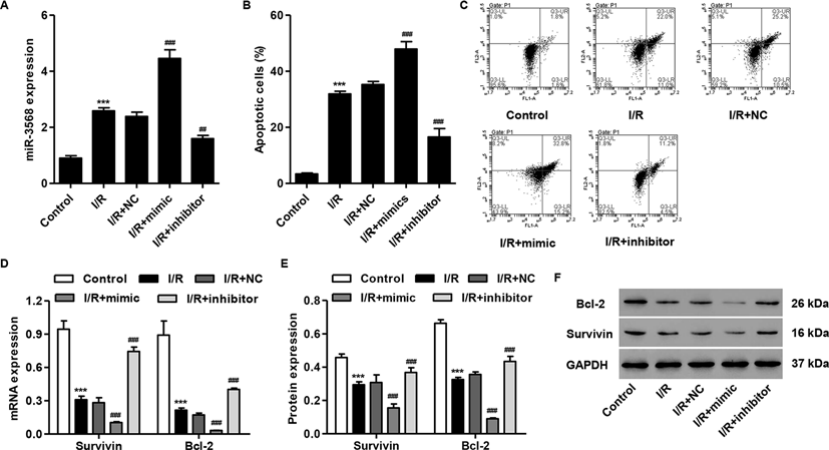
miR-3568 mimic induced cell apoptosis and inhibited the expression of Survivin and Bcl-2 in simulated I/R-induced H9C2 cardiomyocytes. H9C2 cardiomyocytes were transfected with miR-3568 mimic or inhibitor and then simulated I/R for 24 h. **(A)** The expression of miR-3568 was measured by quantitative real-time PCR (n = 3). **(B, C)** The cell apoptosis was measured by flow cytometry (n = 3). The Survivin and Bcl-2 expressions were measured by quantitative real-time PCR (**D**; n = 3) and western blot (**E**, **F**; n = 3). Statistical analyses were carried out using one-way **(A, B)** or two-way **(D, E)** analysis of variance followed by Bonferroni post-test. ***P < 0.001 compared with control. ^##^P < 0.01, ^###^P < 0.001 compared with I/R + negative control (NC).

To examine our hypothesis, simulated I/R-induced H9C2 cardiomyocytes were transfected with miR-3568 mimic or inhibitor, respectively. As shown in **Figure 2A**, miR-3568 mimic transfection significantly increased the miR-3568 expression by 86.4%, and miR-3568 inhibitor transfection significantly decreased the miR-3568 expression by 34.4%, at 24 h post-simulated I/R in H9C2 cardiomyocytes compared with NC. Flow cytometry analysis demonstrated that simulated I/R-induced H9C2 cardiomyocytes showed 8.7-fold increase in the cell apoptosis compared with control. Whereas, miR-3568 mimic significantly increased simulated I/R-induced cell apoptosis by 33.2%, but miR-3568 inhibitor significantly decreased that by 53.9%, compared with NC (**Figures 2B, C**). Western blot and quantitative real-time PCR analysis indicated that simulated I/R-induced H9C2 cardiomyocytes showed decreased Survivin and Bcl-2 expression, which was exacerbated by miR-3568 mimic and was reversed by miR-3568 inhibitor (**Figures 2D–F**).

### miR-3568 Mimic Enhanced Simulated I/R-Induced Activation of MAPKs, AKT, and STAT3 Signaling

**Figure 3:**
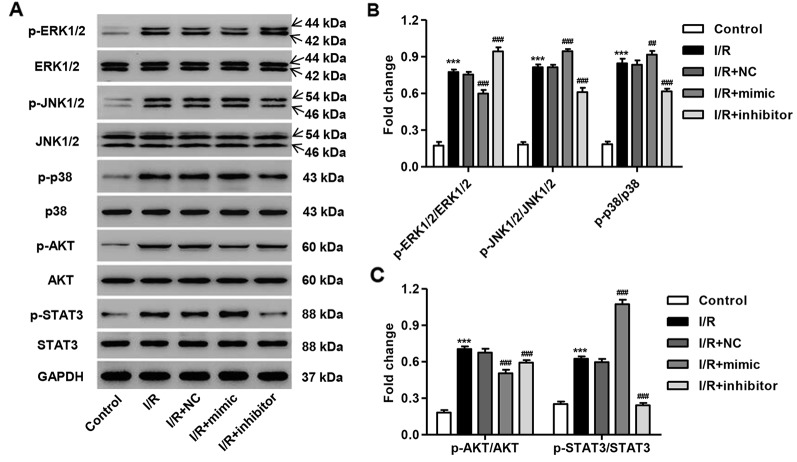
miR-3568 mimic regulated the MAPKs, AKT, and STAT3 signaling in simulated I/R-induced H9C2 cardiomyocytes. H9C2 cardiomyocytes were transfected with miR-3568 mimic or inhibitor and then simulated I/R for 3 h. **(A–C)** The expression of p-ERK1/2, ERK1/2, p-JNK1/2, JNK1/2, p-p38, p38, p-AKT, AKT, p-STAT3, and STAT3 was measured by western blot (n = 3). Statistical analyses were carried out using two-way analysis of variance followed by Bonferroni post-test. ***P < 0.001 compared with control. ^##^P < 0.01, ^###^P < 0.001 compared with I/R + NC.

The activation of several protein kinases, including mitogen-activated protein kinases (MAPKs), AKT, and STAT3, is involved in cell apoptosis. We, therefore, measured the phosphorylation of these proteins in simulated I/R-induced H9C2 cardiomyocytes with miR-3568 mimic or inhibitor transfection. As shown in **Figures 3A–C**, the phosphorylation level of p38, JNK1/2, ERK1/2, AKT, and STAT3 protein was significantly increased at 3 h post-simulated I/R in H9C2 cardiomyocytes compared with control. miR-3568 mimic significantly promoted the phosphorylation of JNK1/2, p38, and STAT3, but inhibited that of ERK1/2 and AKT, in H9C2 cardiomyocytes following simulated I/R, which were reversed by miR-3568 inhibitor (**Figures 3A–C**). However, the p38, JNK1/2, ERK1/2, AKT, and STAT3 protein expression was not obviously changed in H9C2 cardiomyocytes in different conditions. Moreover, compared with other protein kinases, p-STAT3 expression was the most significantly regulated by miR-3568, therefore, suggesting that STAT3 signaling may play an important role in the regulation of miR-3568 in simulated I/R-induced apoptosis.

### TRIM62 Was a Target of miR-3568

**Figure 4:**
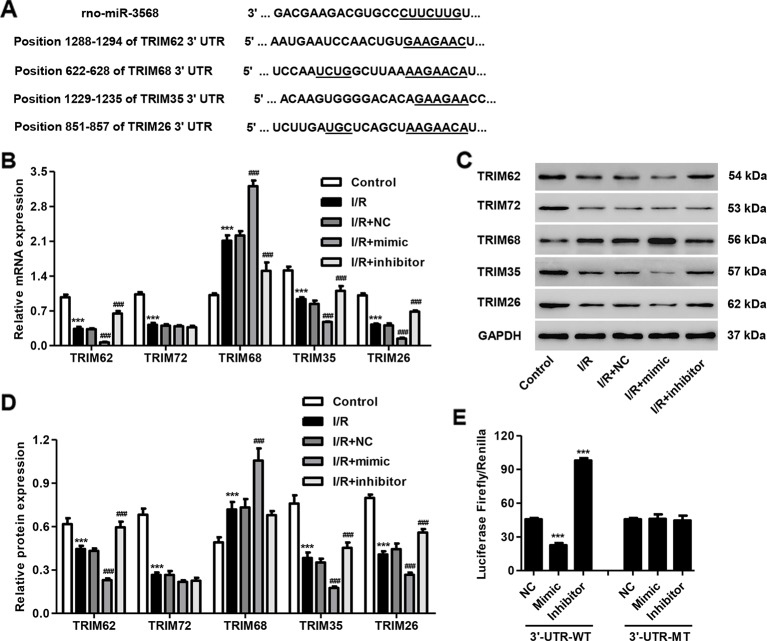
TRIM62 is a target of miR-3568. H9C2 cardiomyocytes were transfected with miR-3568 mimic or inhibitor and then simulated I/R for 24 h. **(A)** Complementary miR-3568 binding sequences in the 3′-UTR of TRIM62, TRIM68, TRIM35, and TRIM26. The expression of TRIM62, TRIM72, TRIM68, TRIM35, and TRIM62 was measured by quantitative real-time PCR (**B**; n = 3) and western blotting (**C**, **D**; n = 3). **(E)** Luciferase activity (Firefly and Renilla) was measured with the Dual-Luciferase Reporter assay (n = 3). Statistical analyses were carried out using one-way **(E)** or two-way **(B, D)** analysis of variance followed by Bonferroni post-test. ***P < 0.001 compared with control or NC. ^###^P < 0.001 compared with I/R + NC.

Previous study has shown that TRIM72 protects myocardium following I/R injury to the heart (Corona et al., 2014; Liu et al., 2015). Some paralogs and SIMAP similar genes for TRIM72 calculated by GeneCards (http://www.genecards.org/), including TRIM62, TRIM68, TRIM35, and TRIM26, are putative targets of miR-3568 using TargetScan (http://genes.mit.edu/targetscan/), and were therefore selected in our following experiments (**Figure 4A**). As shown in **Figures 4B–D**, TRIM62, TRIM72, TRIM35, and TRIM26 expression was decreased in simulated I/R-induced H9C2 cardiomyocytes. Moreover, miR-3568 mimic significantly decreased the expression of TRIM62, TRIM35, and TRIM26, while miR-3568 inhibitor demonstrated an inverse effect. Considering the most significant alternation in TRIM62 expression in response to miR-3568, Dual-Luciferase Reporter assay further confirmed that TRIM62 was a target of miR-3568 (**Figure 4E**). These data indicate that miR-3568 may regulate simulated I/R-induced cell apoptosis through targeting TRIM62.

### TRIM62 Interacted With p-STAT3 and Induced p-STAT3 Ubiquitination

**Figure 5:**
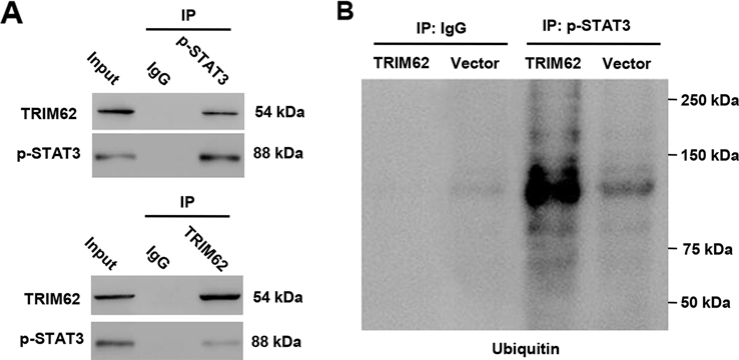
TRIM62 interacts with and induces ubiquitination of p-STAT3. H9C2 cardiomyocytes were transduced with or without pLVX-Puro-TRIM62 or blank pLVX-Puro vector. **(A, B)** TRIM62 or p-STAT3 was immunoprecipitated and immunoblotted with the indicated antibodies (n = 3).

In view of the regulation of miR-3568 in the activation of STAT3 in simulated I/R-induced H9C2 cardiomyocytes, we suggest that TRIM62 may as an E3 ubiquitin ligase participant in this progression. Our data according to the co-immunoprecipitation and ubiquitination analysis found that TRIM62 interacted with p-STAT3 and induced p-STAT3 ubiquitination in H9C2 cardiomyocytes (**Figures 5A, B**). These results indicate that miR-3568 may activate STAT3 signaling through targeting TRIM62, inhibiting p-STAT3 ubiquitination.

### TRIM62 Overexpression or AG490 Treatment Inhibited Simulated I/R- and miR-3568 Mimic-Induced Cell Apoptosis and STAT3 Activation

**Figure 6:**
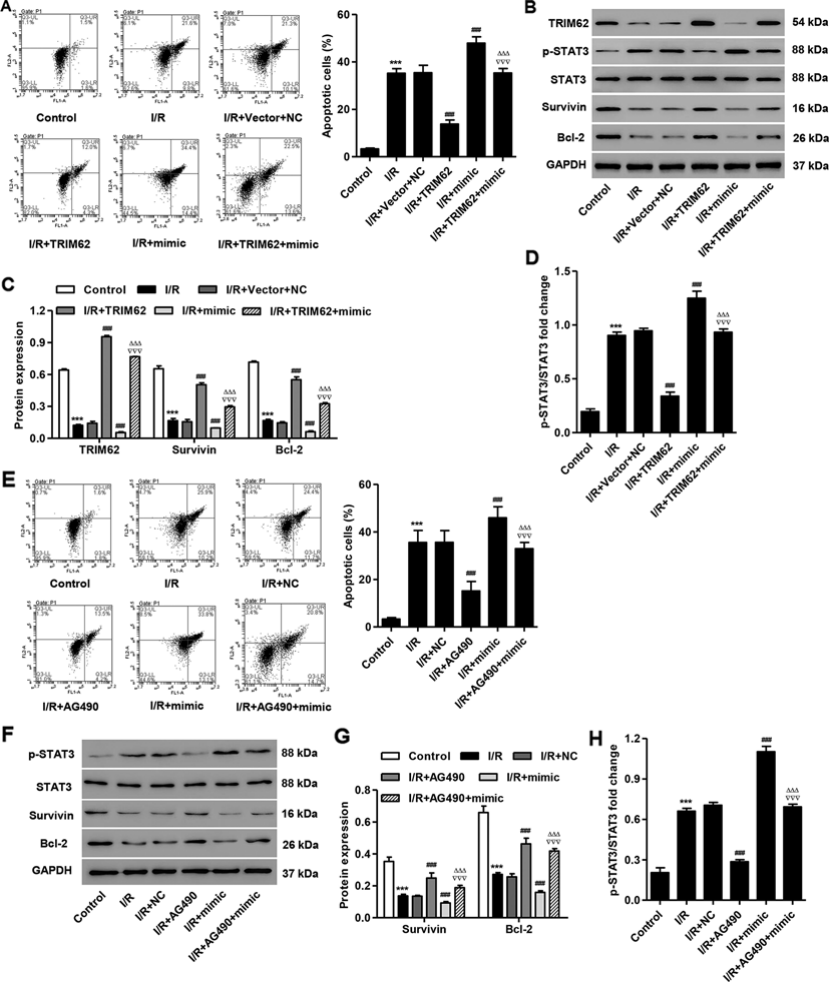
TRIM62 overexpression or AG490 treatment inhibits simulated I/R- and miR-3568 mimic-induced apoptosis and STAT3 activation in H9C2 cardiomyocytes. After H9C2 cardiomyocytes transduced with pLVX-Puro-TRIM62 and/or transfected with miR-3568 mimic and then simulated I/R, the cell apoptosis was measured by flow cytometry (**A**; n = 3) and the Survivin, Bcl-2, p-STAT3, and STAT3 expression was measured by western blot (**B–D**; n = 3). After H9C2 cardiomyocytes treated with AG490 (10 µM) and then simulated I/R for 24 h, the cell apoptosis was measured by flow cytometry (**E**; n = 3) and the Survivin, Bcl-2, p-STAT3, and STAT3 expression was measured by western blot (**F–H**; n = 3). Statistical analyses were carried out using one-way **(A, C, E, H)** or two-way **(D, G)** analysis of variance followed by Bonferroni post-test. ***P < 0.001 compared with control. ^###^P < 0.001 compared with I/R + Vector + NC. ^ΔΔΔ^P < 0.001 compared with I/R + TRIM62. ^∇∇∇^P < 0.001 compared with I/R + mimic.

To further confirm the function of TRIM62 in simulated I/R-induced cell injury, pLVX-Puro-TRIM62 was transduced into the simulated I/R-induced H9C2 cardiomyocytes. We found that TRIM62 overexpression significantly inhibited simulated I/R- and miR-3568 mimic-induced cell apoptosis (**Figure 6A**). Western blotting showed that TRIM62 overexpression significantly inhibited simulated I/R- and miR-3568 mimic-induced activation of STAT3 and decreases in the Survivin and Bcl-2 expression (**Figures 6B–D**). Moreover, AG490, a selective inhibitor of JAK2/STAT3, significantly inhibited I/R- and miR-3568 mimic-induced cell apoptosis, activation of STAT3, and decrease in the Survivin and Bcl-2 expression (**Figures 6E–H**). Our findings indicate that miR-3568 regulates cell apoptosis through TRIM62/STAT3 signaling pathway in simulated I/R-induced H9C2 cardiomyocytes.

### miR-3568 Inhibitor Pre-Treatment Inhibited Cardiac I/R Injury *In Vivo*

**Figure 7:**
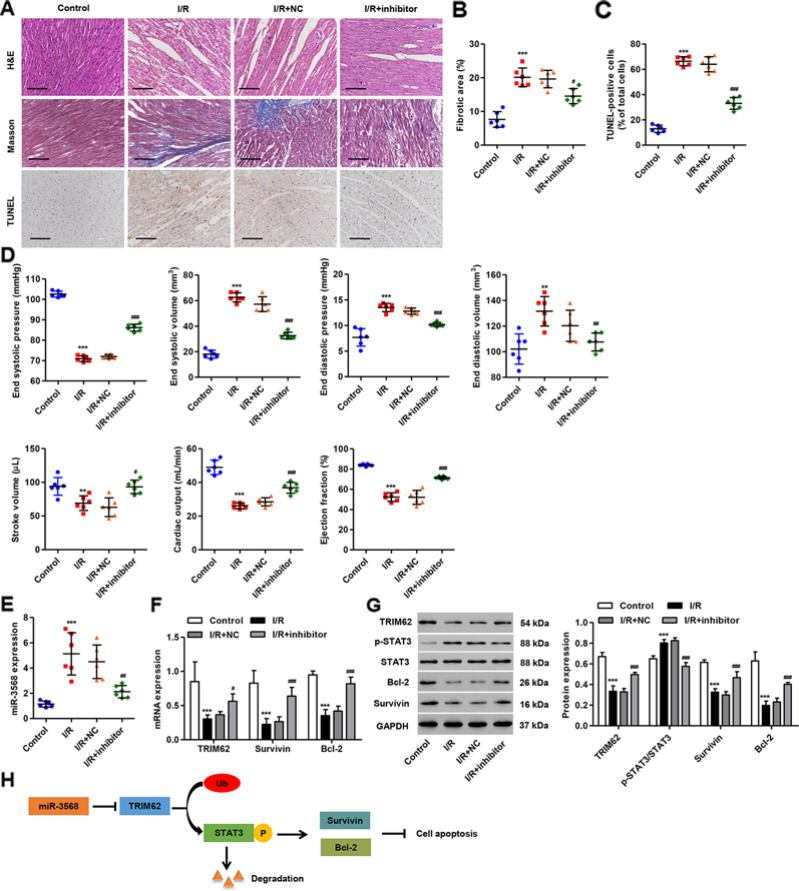
miR-3568 inhibitor pre-treatment reduces cardiac I/R injury *in vivo*. miR-3568 inhibitor or its NC was injected into the left ventricular anterior wall of rats 24 h before I/R. **(A)** Representative photomicrographs of hematoxylin and eosin (HE), Masson, and terminal-deoxynucleoitidyl transferase mediated nick end labeling (TUNEL)-stained myocardium harvested 2 weeks post-I/R (n = 6). Quantization of fibrotic area (**B**; n = 6) and TUNEL-positive cells (**C**; n = 6). Cardiac functional parameters 2 weeks post-I/R were shown in **(D)** (n = 6). The expression of miR-3568, TRIM62, Survivin, Bcl-2, p-STAT3, and STAT3 in myocardium harvested 24 h post-I/R was measured by quantitative real-time PCR (**E**, **F**; n = 6) and western blotting (**G**; n = 6). **(H)** Schematic representation of the regulation of cell apoptosis by miR-3568/TRIM62/p-STAT3. Scale bar: 100 µm. Statistical analyses were carried out using one-way **(B–E)** or two-way **(F**, **G)** analysis of variance followed by Bonferroni post-test. **P < 0.01, ***P < 0.001 compared with control. ^#^P < 0.05, ^##^P < 0.01, ^###^P < 0.001 compared with I/R + NC.

To further investigate the effect of miR-3568 on I/R-induced cardiac injury, the *in vivo* I/R model was established in rats. Histological assessment showed that cardiomyocyte injury, inflammatory cell infiltration, and cardiac cell apoptosis and fibrosis were observed in myocardium ischemic border area of I/R rats, which were relieved with miR-3568 inhibitor pre-treatment (**Figures 7A–C**). Moreover, I/R also caused increases in end systolic volume, end diastolic volume and end diastolic pressure, but reductions in end systolic pressure, stroke volume, cardiac output, and ejection fraction (**Figure 7D**). However, miR-3568 inhibitor pre-treatment could improve I/R-induced cardiac function. Furthermore, miR-3568 expression was remarkably upregulated in rat hearts following I/R. Transfection of miR-3568 inhibitor into the myocardium could significantly decrease miR-356 expression (**Figure 7E**). miR-3568 inhibitor pre-treatment significantly inhibited I/R-induced increase in the STAT3 activation and decrease in the TRIM62, Survivin, and Bcl-2 expression (**Figures 7F, G**). These data further supported the findings in I/R model H9C2 cardiomyocytes.

## Discussion

Apoptosis may play an important role in the process of myocardial I/R injury. Inhibition of myocardial apoptosis can alleviate myocardial I/R injury. The apoptosis occurrence in myocardial I/R injury is associated with the release of oxygen free radicals, intracellular calcium overload and mitochondrial damage (Chao et al., 2006; White et al., 2016; Zhang et al., 2016), and reperfusion will activate endonuclease, resulting in DNA fragmentation and inducing apoptosis in cardiomyocytes (Chang et al., 2013). Given the importance of miRNAs in cell proliferation and apoptosis by posttranscriptionally regulating gene expression, we established a cardiac I/R model in H9C2 cardiomyocytes *in vitro* and in rats *in vivo* to confirm our hypothesis that miR-3568 may be involved in I/R-induced cardiac injury. Our results demonstrated that up-regulated expression of miR-3568 was found in simulated I/R-induced H9C2 cardiomyocytes in a time-dependent manner. Overexpression of miR-3568 by transfection of H9C2 cardiomyocytes with miR-3568 mimic significantly increased the simulated I/R-triggered cell apoptosis and activation of MAPKs, AKT, and STAT3 signaling. Inhibition of miR-3568 by transfection of H9C2 cardiomyocytes with miR-3568 inhibitor (anti-miR-3568) showed an inverse effect, which was further confirmed *in vivo* using I/R rat models. TRIM62 interacted with p-STAT3 and induced the ubiquitination of p-STAT3, which may involve in the regulation of miR-3568 in cardiac I/R injury (**Figure 7H**).

It should be noted that aberrant expression of miR-3568 have been demonstrated in alcoholic steatohepatitis (Chen et al., 2013), chronic kidney disease (Chaturvedi et al., 2015), and osteoarthritis (Chen et al., 2016). In the present study, we demonstrated that miR-3568 expression was up-regulated at 6, 12, and 24 h post-simulated I/R in the H9C2 cardiomyocytes. Similar to our findings, a microarray analysis revealed that at 24 h post-spinal cord I/R injury, miR-3568 was up-regulated compared to its level in sham-operated controls, while the function role of miR-3568 was not demonstrated (Li et al., 2015b; Chen et al., 2019a). Recently, our study showed that miR-3568 overexpression significantly enhanced simulated I/R-induced cell apoptosis and decrease in the expression of Survivin and Bcl-2, and miR-3568 inhibitor significantly inhibited I/R-induced cardiac injury both *in vitro* and *in vivo*. Survivin and Bcl-2 are correlated with the reduction of cardiomyocyte apoptosis and heart ischemic preconditioning (Kaga et al., 2006). Similarly, miR-3568 has been found to be associated with apoptotic process and PPAR signaling pathway that is involved in the induction of apoptosis, suggesting its important role in apoptosis (Chen et al., 2013). These data indicate that miR-3568 overexpression may have pro-apoptotic potentials through the down-regulation of Survivin and Bcl-2 expression. However, the direct interaction between them needs further investigation.

In addition, the target genes of miR-3568 were involved in the MAPK signaling pathway which has been shown to be involved in apoptosis (Chen et al., 2013). MAPKs, including p38 MAPK, JNK1/2 and ERK1/2, AKT, and STATs signaling, are of great importance in response to various stumuli, such as I/R injury. The activation of ERK1/2, JNK1/2, p38 MAPK, AKT, and STAT3 was observed at 30 min post-I/R in cardiomyocytes (Zhang et al., 2011). Similar to our findings that simulated I/R treatment for 3 h significantly activated the p38 MAPK, JNK1/2, ERK1/2, AKT, and STAT3 signaling, with the highest level of STAT3 signaling. However, the contrary results were also demonstrated in other studies. ERK1/2 and AKT phosphorylation were decreased in HUVECs at 24 h post-I/R, while the phosphorylation of JNK1/2 and p38 was not obviously altered in response to simulated I/R (Zhang et al., 2009). Song et al. (2014) found that ERK1/2 signaling was inactivated, JNK1/2 and p38 signaling were activated, and AKT signaling was not altered in mice and in neonatal cardiomyocytes 24 h after I/R injury. In addition, AKT and ERK1/2 signaling pathways were activated, but STAT3 signaling was inactivated by I/R for 24 h in mice (Jiang et al., 2015). These results suggest that the phosphorylation level of ERK1/2, JNK1/2, p38 MAPK, AKT, and STAT3 was time-dependently regulated by I/R injury. Furthermore, miR-3568 overexpression significantly increased simulated I/R-induced activation of JNK1/2, p38 MAPK, and STAT3 signaling, while miR-3568 inhibitor showed an inverse effect, indicating that these signaling pathways may involve in the regulation of miR-3568 in simulated I/R induced injury.

Although the results from computational miRNA target prediction algorithms revealed that miR-3568 had 3277 potential targets, previous study showed that TRIM72 can ameliorate I/R induced muscle injury (Corona et al., 2014; Liu et al., 2015). Surprisingly, 13 TRIM proteins were listed in miR-3568 TargetScan results, among which TRIM62, TRIM68, TRIM35, and TRIM26 are the paralogs and SIMAP similar genes for TRIM72, speculating that they may share the function in I/R injury. Therefore, these TRIMs as well as TRIM72, although not predicted for miR-3568 target, was also investigated in our following experiments. We found that, compared with other TRIM proteins, TRIM62 expression was significantly decreased in response to simulated I/R injury and directly regulated by miR-3568. TRIM62 as a RING finger E3 ubiquitin ligase induced ubiquitination of CARD9, involving in fungal infection and intestinal inflammation (Cao et al., 2015). MAGEC2 promotes metastasis in human hepatocellular carcinoma cells by reducing the ubiquitin-mediated proteolysis of p-STAT3 (Song et al., 2017). Therefore we hypothesize that TRIM62 may interact with p-STAT3 and induce the ubiquitination of p-STAT3. It is exciting that these data were confirmed in H9C2 cardiomyocytes. The subcellular localization showed that TRIM protein family were located in cytosol and nucleus, and some TRIM proteins are capable to induce apoptosis, while the others, on the contrary, have an inhibitory effect on this process (Nenasheva and Stepanenko, 2018). TRIM69 regulated cell apoptosis through p53 signaling (Han et al., 2016). TRIM31 overexpression promoted K63-linked polyubiquitination of TRAF2 and sustained the activation of NF-κB, which subsequently activated multiple anti-apoptosis downstream genes (Yu et al., 2018). TRIM59 promoted cell proliferation and inhibited apoptosis through Wnt/β-catenin signaling (Chen et al., 2019b). TRIM14 overexpression could inhibit SHP-1 which inhibits the phosphorylation of STAT3, thus promoting p-STAT3 and inhibiting apoptosis (Hu et al., 2019). Similarly, TRIM62 overexpression or AG490 treatment significantly inhibited miR-3568 mimic-induced cell apoptosis, STAT3 activation, and decrease in the expression of Survivin and Bcl-2. Activation of STAT3 signaling upregulated the expression of various genes involved in cell proliferation and apoptosis such as Bcl-2 and Survivin (Wang et al., 2015). These data indicate that TRIM62 may suppress miR-3568-mediated I/R injury through ubiquitination of p-STAT3.

## Conclusion

In conclusion, our data suggest that up-regulation of miR-3568 expression contributes to cardiac I/R injury. miR-3568 inhibition gains a protective effect against simulated I/R-induced cell apoptosis through increasing TRIM62 expression in H9C2 cardiomyocyte. TRIM62 interacts with p-STAT3 and induces the ubiquitination of p-STAT3, which may involve in the regulation of miR-3568 in cardiac I/R injury.

## Data Availability Statement

The raw data supporting the conclusions of this article will be made available by the authors, without undue reservation, to any qualified researcher.

## Ethics Statement

The present study was performed in strict accordance with the guidelines on ethical care for experimental animals and approved by the Animal Research Committee of Shanghai East Hospital.

## Author Contributions

XL and XW designed the study. XW, Y-sL and X-dW performed the experiments. X-dW and JZ collected, analyzed, and interpreted the data. XL and HZ prepared the manuscript. All authors read and approved the final manuscript.

## Funding

This work was funded by the Key Disciplines Group Construction Project of Pudong Health Bureau of Shanghai (PWZxq2017-05), and the National Natural Science Foundation of China (81960060).

## Conflict of Interest

The authors declare that the research was conducted in the absence of any commercial or financial relationships that could be construed as a potential conflict of interest.
